# Toward a common theory for learning from reward, affect, and motivation: the SIMON framework

**DOI:** 10.3389/fnsys.2013.00059

**Published:** 2013-10-07

**Authors:** Christopher R. Madan

**Affiliations:** Department of Psychology, University of AlbertaEdmonton, AB, Canada

**Keywords:** reward, affect, motivation, movements, emotion, context, arousal, valence

## Abstract

While the effects of reward, affect, and motivation on learning have each developed into their own fields of research, they largely have been investigated in isolation. As all three of these constructs are highly related, and use similar experimental procedures, an important advance in research would be to consider the interplay between these constructs. Here we first define each of the three constructs, and then discuss how they may influence each other within a common framework. Finally, we delineate several sources of evidence supporting the framework. By considering the constructs of reward, affect, and motivation within a single framework, we can develop a better understanding of the processes involved in learning and how they interplay, and work toward a comprehensive theory that encompasses reward, affect, and motivation.

## Introduction

Reward, affect, and motivation are three highly related constructs, but are often investigated in isolation despite using similar experimental procedures. As an example, contextual fear conditioning is a common task used to investigate affective learning in rats. In this task, a rat is kept in a two-compartment chamber. Over time the rat gradually learns that when a tone is presented, the floor of one compartment of the chamber will deliver an electric shock. With respect to the affect construct, this task is described as eliciting a fear-related response (i.e., fleeing or freezing) when the tone occurs. However, this procedure is nearly identical to a conditioned place preference task, where the dependent measure is the proportion of time that the rat spends in each compartment, after the rat has been conditioned with shocks. Here the task is described as measuring motivational effects, e.g., approach vs. avoidance. Furthermore, it is important that an integral aspect of the task is the use of shocks, an aversive stimulus with respect to the reward construct, to elicit learning.

While it is possible to disentangle these constructs experimentally, they often coincide in real-world experiences and can converge and conflict in important ways. Here we briefly define each construct and discuss how they function in concert, as described by the proposed SIMON framework. By discussing the interplay of the constructs, we can lay the foundation for the development of a common theory encompassing reward, affect, and motivation.

## Defining the constructs

Before we can discuss interactions of reward, affect, and motivation, it is important to operationalize the three constructs independently. As the descriptions below are relatively brief, it is suggested to refer to the cited reviews for further details.

### Reward

Reward is the most clearly defined of the three constructs, particularly when viewed from an operant conditioning perspective: an organism learns that by responding (R) to a stimulus (S), an outcome (O) is presented. The outcome can either be appetitive (i.e., elicit an approach response), such as food, or aversive (i.e., elicit avoidance), such as an electric shock. Thus, in this simplified form, reward-based learning can be described as a S–R–O association (i.e., operant conditioning). To clarify the reward construct, it is important to note that while often used interchangeably, “reward” and “reinforcement” do not have identical meanings (White, 1989; Berridge and Robinson, 1998). Specifically, while rewards (i.e., appetitive stimuli) elicit approach responses, reinforcement should be used to describe the increase of responses to a stimulus. For further clarity, we will define “reward” as the construct itself, where outcomes can be either “appetitive” and “aversive,” rather refer to outcomes as being “rewarding” (i.e., appetitive). It is also important to note that many different types of stimuli can be appetitive, such as monetary, food, and erotic stimuli (see Sescousse et al., 2013 for a review), while aversive stimuli usually are either monetary losses or electric shocks. Kirsch et al. (2004) provide a comprehensive discussion on the role of reward-based learning (specifically, conditioning) on cognition.

**Figure 1:**
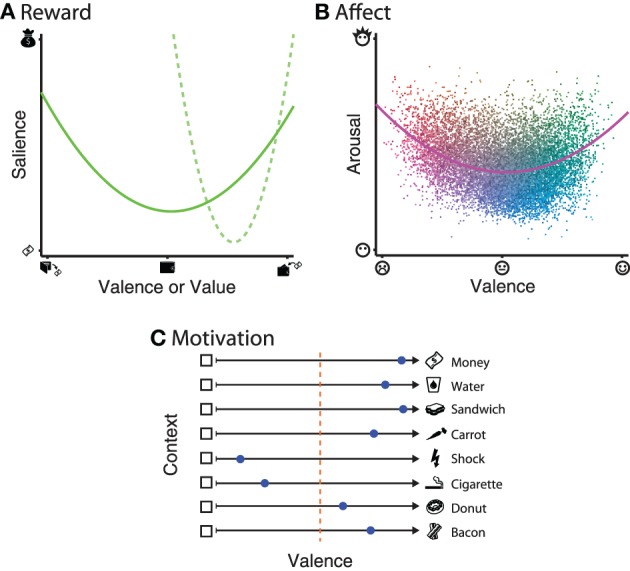
**Illustrations of the dimensionality of each of the three constructs. (A)** Reward: the solid line denotes that reward salience has a quadratic relationship relative to reward valence. Recent results have also shown that this relationship can be observed even when the range of values experienced is constrained to either the gain or loss domain, as in the dashed line. **(B)** Affect: each dot represents a word from a large normative database Warriner et al., (2013). Dot color varies between blue–yellow (based on arousal) and red–green (based on valence), with variability in luminance added to improve item discriminability. The solid line represents a quadratic model fit to all words in the database. **(C)** Motivation: approach–avoidance tendencies are context-dependant, based on not only stimulus itself, but also the current state of the individual (e.g., thirst, hunger) and inter-individual differences (e.g., economics status, smoker, dieter, vegetarian). Individual preference within a given context are denoted by the blue dots, and range from approach (dot closer to the stimulus) to avoid (dot closer to the empty box). The orange dotted line denotes the point of indifference.

It is unarguable that rewards can vary along at least one dimension: value; when gains vs. losses are included, this dimension is often referred to as reward valence. However, recent findings suggest that reward is coded in the brain along two orthogonal dimensions: valence and salience (Figure 1A). Briefly, reward valence ranges from appetitive to aversive. Reward salience is defined by a quadratic relationship relative to value, such that the highest and lowest values experienced are highest in salience. Evidence for separable coding of reward salience is mainly supported by neural activations that correlate with the magnitude of outcomes, independent of the valence (Zink et al., 2004; Jensen et al., 2007; Cooper and Knutson, 2008; Litt et al., 2011). Recent behavioral studies have supported the notion of reward salience, even when the range of experienced outcomes is constrained to only the gain or loss domain [(Ludvig et al., 2013; Madan and Spetch, 2010); e.g., Figure 1A, dashed line].

Neuroimaging results suggest that reward-related activations in the medial orbitofrontal cortex, rostral anterior cingulate cortex, and dorsal posterior cingulate correspond to reward value, while activations in the dorsal anterior cingulate cortex and insula correspond to reward salience (Litt et al., 2011). Activations in the ventral striatum, and particularly the nucleus accumbens, correspond to a mixture of reward value and salience, with salience playing a stronger role. There is also evidence of dissociable brain regions associated with gain vs. loss outcomes (Yacubian et al., 2006; Eppinger et al., 2013).

### Affect

Affect can be defined as the conscious experience of emotions (Panksepp, 2000; Yik et al., 2011), though affect and emotion are often used synonymously [also see Kleinginna and Kleinginna (1981a), Lang (2010), and Izard (2010), for in-depth definitions of emotion]. To describe the affective space, Russell, (1980) proposed the circumplex model of affect (also see Yik et al., 2011), which suggests that affect is comprised of two orthogonal dimensions: valence and arousal. Valence ranges from unpleasant to pleasant, while arousal ranges from bored to excited. The orthogonality of these two dimensions is also supported by neuroimaging results (Kensinger and Corkin, 2004; Posner et al., 2009): the valence-specific network was associated with the insula, the arousal-specific network with the medial prefrontal cortex and posterior cingulate, while both networks included the dorsolateral prefrontal cortex, anterior cingulate cortex, and the amygdala.

Within an experimental setting, words and images are used to elicit affect within the participant, most commonly using the International Affective Picture System (IAPS; Lang et al., 2008) and Affective Norms for English Words (ANEW; Bradley and Lang, 1999; but also see Warriner et al., 2013) databases. While affective states fill the complete circumplex space, stimuli often show a U- or boomerang-shaped distribution [Lang et al., (1998); e.g., Figure 1B].

### Motivation

Here we define motivation primarily based on the hedonistic principle (e.g., Young, 1959; White, 1989): motivation is the process of maximizing pleasure (i.e., appetitive, positive affect) and minimizing pain (i.e., aversive, negative affect). The ends of this motivational valence continuum correspomd to approach and avoidance behavior [see (Young, 1959), Kleinginna and Kleinginna, (1981b), and Elliot and Covington, (2001), for detailed definitions of motivation]. Based on this definition, it is clear that motivation is closely related to reward and affect. Additionally, motivation is intrinsically defined as motor movements, to either approach or avoid a stimulus. This perspective also overlaps highly with the idea of motor affordances (Gibson, 1977; Cisek and Kalaska, 2010).

It is also important to note that motivation incorporates contextual information that may influence stimulus processing outside of what could be explained by reward and affective processing alone [e.g., Berridge, (2004); see Figure 1C]. As an example, money can be used as a appetitive outcome, but an individual's drive to obtain money is not always constant. A simpler example would be one's drive for food and water, both of which are appetitive, but an individual is not always hungry/thirsty and may be over-satiated and temporarily not want to consume more food or water, and thus be not approached. Other stimuli may be generally aversive, such as electric shocks; stimuli that reliably predict shocks will lead to avoidance behavior after sufficient learning. However, there are individual differences in approach–avoidance motivation. For instance, smoking is highly aversive to many, but considered appetitive to some. Foods like donuts and bacon can demonstrate even more inter-individual variability: despite being foods and thus generally appetitive, an individual who is dieting should avoid donuts and a vegetarian would actively avoid the bacon.

### Summary

Reward, affect, and motivation are related constructs. However, all constructs are discrete and dissociable: affect is largely an internal state, whereas a reward is related an outcome to be obtained (i.e., a goal) or avoided. While obtaining the outcome, e.g., food, likely also leads to a positive affective state, these are two dissociable processes, such as in the case of over-satiation. The food is still appetitive, but due to over-satiation, motivation is attenuated and the resulting affective state changes accordingly. Despite the strong intrinsic relationships between the constructs, they do not co-vary together in all situations. Studying these instances of disagreement are critical for the development of a comprehensive theory that encompasses all three constructs.

## The simon framework

**Figure 2:**
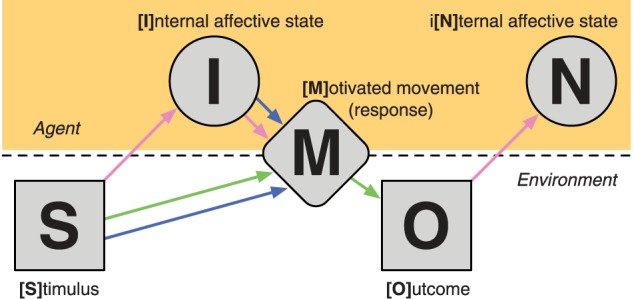
**Illustration of the processes involved in the SIMON framework.** Line arrows correspond to the portions of the framework that are intrinsic to each of the three component constructs: reward (green), affect (pink), and motivation (blue).

While prior studies have discussed portions of their interplay, all three have not been discussed within the same framework. The SIMON framework serves this purpose by delineating a simple framework where the constructs are considered in concert. Here we propose the structure of the SIMON framework and describe prior evidence supporting portions of the framework:

The proposed SIMON framework suggests that after a [S]timulus is presented, it leads to an [I]nternal affective state. The stimulus and the elicited affective state both influence the resulting [M]otivated movement (i.e., response) where the individual responds to the stimulus. Based on the movement (or lack there of), an [O]utcome occurs that then also leads to an i[N]ternal affective state. See Figure 2 for a graphical representation of the SIMON framework. Here we have the three constructs overlaid such that the reward construct is described by the Stimulus–Movement–Outcome (S–M–O) portion of the framework, which is a S–R–O association, i.e., operant conditioning. The affect construct is denoted by the S–I(–M) and O–N portions of the framework, where presented stimuli, including the outcome itself, elicit an affective state, and that this can also lead to a response. The motivation construct is described by the S–I–M portion of the framework, where a stimulus and it's resulting affective state both lead to a motivated movement.

### Evidence for reward → motivation: can reward learning lead to motivated movements?

Learning that a specific action leads to a reward-related outcome is the basis of operant conditioning and much of animal learning as a field (e.g., Balleine and Dickinson, 1998). Additionally, in certain circumstances, this type of learning can lead to the development of superstitious behaviors in both human and non-human animals (e.g., “lucky numbers”; Brown, 1986). An illustration of this type of learning is outlined in (Cardinal et al., 2002, Figure 2), where the behavior resulting from the learning of a stimuli–outcome association are the elicitation of motivated movements, such as lever-pressing and locomotor approach.

Consider two theoretical perspectives that bear on the relation of these two constructs: from a reinforcement learning perspective, an agent's goal is to obtain as much reward (i.e., appetitive stimuli) as possible, by learning from the outcomes of prior actions Woergoetter and Porr, (2008). In other words, seeking of rewards drives motivated movements, a notion supported by a number of studies (e.g., Hikosaka et al., 2008), and supporting the S–M–O portion of the SIMON framework. This rationale is also supported by the motor chauvinist perspective: the purpose of the brain is to produce movements, and sequences of motor actions are constructed to achieve high-level goals, such as acquire appetitive outcomes (Wolpert et al., 2001). Despite markedly different theoretical backgrounds, both perspectives suggest that at a basic level, motor movements are important to acquiring appetitive outcomes and that learning from reward-related experiences can reinforce the production of preceding movements.

### Evidence for affect → motivation: can affect drive motivated movements?

Stimuli can often elicit affective states, and the combination of the stimuli and affective states can lead to a motor response (I–M portion of the SIMON framework). Well-known examples of this phenomena are reflex potentiation (fight-or-flight response) and freezing, and that affective states can influence physiological measures such as pupil dilation, heart rate (e.g., fear bradycardia; Campbell et al., 1997), and skin conductance (Bradley et al., 2008). Furthermore, research has shown that a variety of mammals use similar facial expressions (i.e., movements of the facial muscles) as humans to express positive/appetitive and negative/aversive states (e.g., Berridge, 2004). Lang and Bradley, (2010) discuss evidence that affective stimuli can lead to greater motor potentiation, as measured by neural activation in supplementary motor area, among other brain regions, indicating higher-level cortical involvement, rather than only reflexive motor potentiation due to affect. Also see Carver, (2006) and Harmon-Jones et al., (2013) for further discussions of the coupling between affect and motivation/motor-actions.

### Evidence for reward → affect: can rewards lead to affective states?

Rewards and affect both have important influences on learning, but are often discussed in isolation and use different procedures: studies of reward learning usually use a conditioning-based approach, where the task is learned through trial-and-error with the goal of obtaining the maximal cumulative reward. Studies using affective stimuli usually simply present the affective words/images, though there are instances where affect is conditioned (e.g., Mather and Knight, 2008; Schwarze et al., 2012). Nonetheless, one would expect that that earning an appetitive stimulus should elicit positive affect, whereas a negative outcome, such as a shock, is both aversive and elicits negative affect. This notion also suggested by Rolls, (2000), and would comprise the O–N portion of the framework. Results from Dixon et al., (2010) also support this idea, where physiological measures of arousal were greater for appetitive outcomes (also see Bechara et al., 2005). Brown, (1986) also suggests that arousal can play an important role in problem gambling.

Another line of research supporting the influence of rewards on affect is decision affect theory (Mellers et al., 1997). Here participants were presented with pie charts denoting probabilities of either gaining/losing a specified amount of money or receiving an outcome of $0. After each trial, participants were asked to provide a rating of the emotional state on a Likert scale, ranging from extremely elated (+50) to extremely disappointed (−50), and affective responses were found to follow directly from predictions based on the reward outcome obtained. According to this line of research, in the event of a choice, “elation” is experienced if the outcome exceeds expectations, but “disappointment” is experienced if the outcome is worse than expected (Bell, 1985). If feedback on the forgone/unchosen option is also presented, “regret” is experienced if the chosen option is worse than the unchosen option's outcome, while “relief” is experienced when the chosen outcome led to the better outcome (Bell, 1982; Bryne, 2002). In other words, affective responses are operationalized based on outcomes experienced.

## Evidence supporting the framework

By considering the relations between reward, affect, and motivation, a myriad of behavioral findings support the notion of a single framework. Here we outline a handful of such examples, along with their underlying principles as outlined by the SIMON framework.

### Affective stimuli and motor movements

One of the most straightforward sources of evidence for the SIMON framework is that positive stimuli should be more congruent with an approach response, while negative stimuli should be more congruent with an avoid response. In lexical decision, where participants are presented with a letter-string that may or may not be a word and must judge it's lexicality, participants have been shown to respond relatively faster to positive words and relatively slower to negative words, when compared to neutral words (Estes and Adelman, 2008). Furthermore, in a go/no-go task, participants were slower to respond to images of fearful faces relative to happy faces (Hare et al., 2005). In both instances, participants exhibited a tendency to avoid negative stimuli, in conflict with the instruct to respond (i.e., approach) the stimuli. However, Hare et al., (2005) also reported that when participants are asked to inhibit their responses (i.e., no-go trials), false alarm rates are higher for happy than fearful faces as it is more difficult to suppress the approach response to the positive stimuli. Through similar principles, it has been suggested that approach/avoidance movements can provide information about an animal's affective preferences (Kirkden and Pajor, 2006).

### Motor movements and rewards

Another interesting line of evidence for the SIMON framework is intrinsic relationship between motor actions and rewards. One example of this is motor movements should optimize rewards earned in the task. Consider a reaching task where there are two target areas, each associated with a different reward value, e.g., similar to a dartboard (see Trommershäuser et al., 2008; Cisek, 2012). Participants have been found to aim for a point that would maximize their earnings and minimize potential losses, while also accounting for variability in precision.

A second interaction of motor and rewards can be observed in the influence of medication to treat motor dysfunction on reward-related behavior. It is known that Parkinson's patients taking dopamine agonists are more likely to develop problem gambling behavior (Dodd et al., 2005), a result found to generalize to other disorders also treated with dopamine agonists (e.g., d'Orsi et al., 2011). A likely cause for this interaction is that even though both gain outcomes and motor movements normally rely on the phasic release of dopamine (e.g., Steinberg et al., 2013), dopamine agonists increase the tonic level of dopamine, still leaving a dysregulation of the dopamine system.

### Conflict in affect and reward to improve executive control

Another source of support for the SIMON framework is the use of affective stimuli that conflict with a reward. For instance, cigarette packages in North America often depict graphical images of the negative consequences of smoking, and have been shown to help individuals quit smoking (Farrelly et al., 2012). Extending this to food stimuli, Veling et al., (2011) presented participants with palatable food images in a go/no-go task, but preceded the food images with affective faces. Images of fearful faces were found to increase response time, suggesting that the conflict between reward and affect can be used to increase impulse control. Hollands et al., (2011) used a similar approach, but instead conditioned individuals to form associations between snack images and aversive bodily images (e.g., obese individuals) and found that the intervention improved healthy food choices relative to a control group. Similar interventions have also been used to treat phobias (e.g., Hekmat and Vanian, 1971).

## Conclusion

In the laboratory we aim to isolate a single construct and research it experimentally. However, in the real world learning is influenced by a multitude of concurrent effects that can be closely inter-related. By considering the constructs of reward, affect, and motivation within a single framework, we can work toward a better understanding of the processes involved in learning and provide an opportunity to refine the definitions of each of the component constructs. Finally, by considering the interplay of these three constructs, several current lines of research can be predicted in a common framework, and we can begin to work toward a comprehensive theory that encompasses reward, affect, and motivation.

### Conflict of interest statement

The author declares that the research was conducted in the absence of any commercial or financial relationships that could be construed as a potential conflict of interest.
